# A Comparison of Tissue Property Values Estimated Using Conventional Cardiac MRF and MT‐Cardiac MRF

**DOI:** 10.1002/mrm.70470

**Published:** 2026-07-01

**Authors:** Sydney Kaplan, Zexuan Liu, Jesse Hamilton, Shaihan Malik, Chaitanya Madamanchi, Venkatesh Murthy, Scott Swanson, Nicole Seiberlich

**Affiliations:** ^1^ Department of Biomedical Engineering University of Michigan Ann Arbor Michigan USA; ^2^ Department of Radiology University of Michigan Ann Arbor Michigan USA; ^3^ Department of Imaging Physics and Engineering King's College London London UK; ^4^ Frankel Cardiovascular Center, Division of Cardiovascular Medicine, Department of Internal Medicine University of Michigan Ann Arbor Michigan USA

**Keywords:** cardiac MRI, magnetization transfer, MR fingerprinting, multiparametric mapping

## Abstract

**Purpose:**

To evaluate the impact of explicitly modeling Magnetization Transfer (MT) during reconstruction of cardiac Magnetic Resonance Fingerprinting (cMRF) maps on T_1_ and T_2_ quantification.

**Methods:**

cMRF data were reconstructed using two dictionaries: a conventional cMRF dictionary that ignores MT processes, and an MT‐cMRF dictionary modeling *T*_1_ and *T*_2_ of the free water pool (*T*_1f_ and *T*_2f_, respectively), as well as the bound pool fraction (BPF) as free parameters. Measurements which include MT processes were assessed in simulation and phantom to ensure that *T*_1f_, *T*_2f_, and BPF could accurately be measured using cMRF. cMRF data were then collected in 14 healthy subjects to measure *T*_1_/*T*_1f_, *T*_2_/*T*_2f_, and BPF in normal myocardium. Finally, cMRF data were acquired in five patients with known or suspected myocardial scar at 1.5T, and tissue property maps both ignoring and including MT were generated. Linear discriminant analyses (LDA) were performed to assess differences in tissue property values between scar and remote myocardium.

**Results:**

MT‐cMRF showed excellent agreement with ground truth simulations (RMS error = 2.4% ± 3.9%) and phantom measurements (*R* ^2^ = 0.99). Values measured with MT‐cMRF in healthy subjects (*T*_1f_ = 1137 ± 72 ms, *T*_2f_ = 42.8 ± 4.2 ms, BPF = 8.2% ± 1.2%) were significantly different than conventional cMRF (*T*_1_ = 947 ± 75 ms, *T*_2_ = 41.5 ± 3.9 ms). *T*_1f_/*T*_2f_ and *T*_2f_/BPF from MT‐cMRF demonstrated higher LDA accuracy (82.4% and 86.1%) in discriminating scar from healthy tissue than *T*_1_ and *T*_2_ from conventional cMRF (61.8%).

**Conclusion:**

Incorporating MT modeling into cMRF results in improved *T*_1f_ and *T*_2f_ quantification compared to conventional modeling and enables BPF measurement. Native MT‐cMRF *T*_1f_, *T*_2f_, and BPF values showed differences between scar and healthy myocardium.

## 1 Introduction

Quantitative myocardial tissue characterization with cardiac magnetic resonance (CMR) has proven valuable for detecting and differentiating between various myocardial diseases. Elevations in *T*_1_ and *T*_2_ can serve as indicators of fibrosis, infarction, edema, and other myocardial abnormalities, making accurate and reliable quantification essential for proper diagnosis and management [1, 2, 3, 4]. Existing mapping techniques for *T*_1_ and *T*_2_, including both MOLLI and cardiac Magnetic Resonance Fingerprinting (cMRF), involve fitting acquired data to a signal model; however, these approaches often rely on single‐pool approximations which assume homogeneous tissue composition and effectively ignore magnetization transfer (MT) processes in order to simplify the modeling [5, 6, 7]. On the other hand, recent studies have suggested that neglecting MT effects may reduce the accuracy of tissue property values obtained with both conventional and MRF‐based measurements [8, 9, 10]. In contrast, using a more comprehensive two‐pool model that incorporates both free and macromolecular‐bound water pools, as well as their exchange dynamics, may enable tissue property estimation that better reflects the underlying tissue dynamics. This is particularly important for disease processes such as myocardial scarring, which are characterized by increased levels of collagen and other macromolecules that can alter MT dynamics and where failure to account for MT can introduce bias into *T*_1_ and *T*_2_ measurements [11]. Adopting a two‐pool model which incorporates MT effects is therefore expected to improve the accuracy and reliability of *T*_1_ and *T*_2_ measurements. Moreover, studies have suggested that measurements of MT properties, such as the bound pool fraction (BPF) and magnetization exchange rate, may provide valuable insight into changes in macromolecular content associated with scar tissue without the need for gadolinium‐based contrast agents (GBCAs) [11, 12, 13, 14, 15, 16]. Consequently, combining MT quantification with traditional *T*_1_ and *T*_2_ measurements may potentially provide a more sensitive non‐invasive marker for myocardial scar than using a single measurement alone.

MRF offers unique advantages for myocardial mapping, enabling simultaneous acquisition of multiple, co‐registered parametric maps, including *T*_1_, *T*_2_, and quantitative MT tissue properties [6, 17, 18, 19]. The approach of collecting multiple maps simultaneously can significantly streamline the workflow in high‐volume clinical environments by decreasing overall scan time and the number of required breath‐holds. The co‐registered nature of the maps derived from MRF can be used to assess tissue composition on a per‐voxel basis, enabling small focal abnormalities to be studied. Additionally, as MRF can be used to model the influence of multiple tissue properties on the MRI signal, the maps that result from MRF processing are expected to be more accurate and reproducible than quantification methods that use simpler but incomplete signal models. In this study, the effect of explicitly modeling MT in the dictionary used to reconstruct tissue property maps from cMRF data is evaluated first for accuracy in phantom measurements, then in healthy subjects to establish the impact of including MT processes into the cMRF reconstruction. cMRF data were then collected in patients with myocardial scar to assess whether native MT tissue properties were different in areas of scar and normal myocardium. This study offers preliminary insights into the potential benefits of MT‐cMRF for improved accuracy of MRF‐based tissue property quantification.

## 2 Methods

### 2.1 Acquisition and Reconstruction

A previously described 2D cMRF protocol designed for quantifying *T*_1_ and *T*_2_ in the heart was used in this study [6, 18]. Data were acquired in an ECG‐triggered acquisition across 15 heartbeats in diastole during breath‐holding. The data collection phase in each heartbeat consisted of a preparation pulse followed by a series of 45 excitations with flip angles ranging from 4° to 25° in a ramp‐up pattern. Preparation pulses were deployed in a five‐heartbeat (HB) arrangement (HB 1: inversion, TI = 21 ms; HB 2: no preparation; HB 3: *T*_2_, TE = 30 ms; HB 4: *T*_2_, TE = 50 ms; HB 5: *T*_2_, TE = 80 ms), which was repeated three times. Data were acquired using a fast imaging with steady‐state precession (FISP) readout along a highly undersampled (*R* = 48) spiral trajectory, and after each TR an 8*π* phase twist spoiler gradient was applied. Acquisition parameters include voxel size = 1.6 × 1.6 mm^2^, slice thickness = 8 mm, FOV = 300 mm^2^, constant TR/TE 5.4/1 ms, RF pulse duration = 800 μs, and time bandwidth product 2.

**FIGURE 1:**
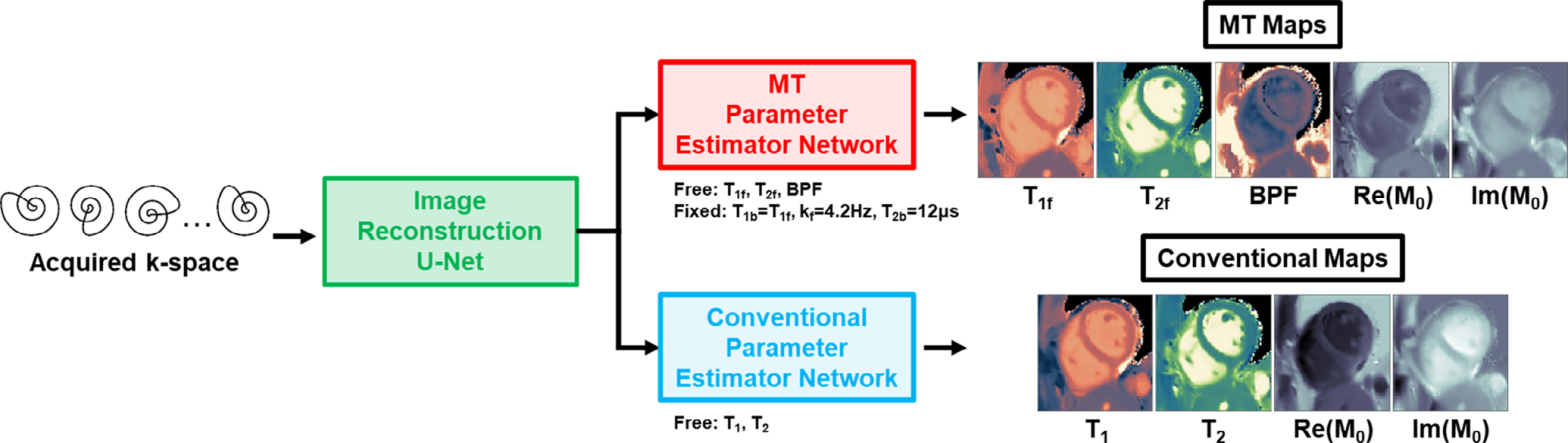
The reconstruction pipeline used to generate *T*_1_/*T*_1f_, *T*_2_/*T*_2f_, and BPF maps from cMRF data.

The reconstruction pipeline for generating tissue property maps from the cMRF data is shown in Figure 1. The maps were reconstructed in two ways to assess the impact of modeling MT: first using a dictionary which ignored MT processes (resulting in maps for *T*_1_ and *T*_2_), and second using a dictionary including MT (resulting in maps for *T*_1f_, *T*_2f_, and BPF); adding MT into the signal model is expected to improve accuracy in tissue property measurements. A Deep Image Prior (DIP) [20, 21] reconstruction was used in both cases to improve overall map quality [22, 23] (see Supporting Information [SI] *Reconstruction Comparison* for comparison against other reconstruction methods). The conventional cMRF dictionary, which does not include MT, was generated via extended phase graph (EPG) signal modeling, assuming that all of the signal in a voxel arises from a single pool of water with a single value of *T*_1_ and *T*_2_. The second dictionary uses signal modeling that includes MT effects using the EPG‐X framework [24]; this approach will be referred to as MT‐cMRF. Here, magnetization exchange between free water and bound water pools is modeled, and the tissue properties included in this model are the *T*_1_ and *T*_2_ of the free water (*T*_1f_, *T*_2f_), the *T*_1_ and *T*_2_ of the bound water (*T*_1b_, *T*_2b_), the rate of MT between the pools (*k*_f_) and the relative size of the bound pool (BPF), which defines the sizes of *M*_0f_ and *M*_0b_ that are used to scale the signal contributions from each pool. In this work, a fixed rate of MT between pools (*k*_f_ = 4.2Hz^15^), *T*_1f_ = *T*_1b_, and *T*_2b_ = 12 μs were assumed based on the literature. The tissue properties to be mapped were thus *T*_1f_, *T*_2f_, and the relative size of the bound pool (BPF). The structure of the DIP follows previously described work [20, 21], leveraging untrained neural networks, a forward model of the cMRF imaging process, and signal modeling that either ignores or includes MT effects to generate subspace images and parameter maps.

### 2.2 Simulations

Several simulations were performed to assess the effect of MT on cMRF measurements. Simulations were conducted using the XCAT phantom [25] with a simulated heart rate of 60 bpm with tissue property values assigned to each tissue ranging from *T*_1f_ 250–1500 ms, *T*_2f_ 20–155 ms, and BPF 4%–10%. Two cases were evaluated: (Case 1) healthy myocardium (*T*_1f_ = 1000 ms, *T*_2f_ = 44 ms, and BPF = 8%), and (Case 2) myocardium with a small lesion (15 pixels, *T*_1f_ = 1100 ms, *T*_2f_ = 50 ms, and BPF = 6%) [15, 26, 27]. Signal evolutions for each voxel were computed using the EPG‐X framework, using the assumptions stated above for *T*_1b_, *T*_2b_, and *k*_f_, and the *T*_1f_, *T*_2f_, and BPF values assigned to each voxel, using the cMRF sequence parameters. Simulations included coil sensitivity profiles for a simulated eight‐channel receiver coil as well as the undersampled spiral k‐space readout. Since cMRF data are acquired during diastole under breathholding, no motion was included in the simulations. Data were reconstructed using a DIP approach that included the undersampled k‐space data, coil sensitivity maps, and spiral trajectory information along with either the dictionary generated using conventional modeling or MT modeling. The accuracy of the maps was assessed by computing the root mean squared error (RMSE) against the ground truth maps. See SI: *MT Encoding in the cMRF Fingerprints* and *XCAT Simulations* for additional simulations to assess the strength of MT encoding in the cMRF sequence.

### 2.3 Phantom Study

The ISMRM/NIST MRI system phantom [28] and an MT phantom (see SI: *MT Phantom Preparation*) were imaged at 1.5 T (MAGNETOM Sola, Siemens Healthineers) to evaluate the accuracy of MT‐cMRF. Data were acquired with a 20‐channel head coil in a single slice through the center of the *T*_2_ layer of the NIST phantom and central slice for the MT phantom using a simulated ECG with a heart rate of 60 bpm. cMRF data were reconstructed using both conventional and MT modeling. Accuracy was assessed using linear regression against reference values.

### 2.4 In Vivo Studies

This study was approved by the University of Michigan Institutional Review Board and informed consent was obtained from all participants. All in vivo data were acquired on a single 1.5 T scanner (MAGNETOM Sola, Siemens Healthineers) using an 18‐channel body coil and 12 channels from the spine array.

#### 2.4.1 Healthy Subjects

Fourteen healthy subjects (age = 34 ± 16 years, 7 female) were scanned using the cMRF sequence in a single, mid‐ventricular, short‐axis slice during diastole across a 15‐heartbeat breath‐hold at expiration. Reference MOLLI [5] and *T*_2_‐prep bSSFP [29] maps were also acquired using the Siemens Myomaps product sequences. cMRF maps were reconstructed using both conventional and MT modeling including subject‐specific dictionaries to account for individual cardiac rhythms obtained from the ECG signal. Myocardial *T*_1_ and *T*_2_ (for reference maps and conventional cMRF) and *T*_1f_, *T*_2f_, and BPF values (for MT‐cMRF) were obtained by computing the mean and standard deviation of these measurements within manually drawn ROIs within the six segments suggested for the mid‐ventricular slice by the American Heart Association (AHA) [30]. Differences between tissue property maps generated using each of the modeling techniques were compared using a paired *t*‐test by segment.

#### 2.4.2 Patients

MRI data from four patients (age 55 ± 19 years, 2 female, one cardiomyopathy, three HCM) receiving GBCA as part of their clinical assessment and one renal patient undergoing a contrast‐free research scan were collected. Reference *T*_1_ and *T*_2_ maps and cMRF data were acquired prior to the administration of GBCA in 3–9 short‐axis slices spanning the ventricles in each patient. Conventional LGE images (11–16 short‐axis slices, 8 mm thickness) were acquired in the four patients that received GBCAs 10 min after injection as specified in each patient's clinical protocol. A *T*_1ρ_ map was acquired using *T*_1ρ_‐cMRF [21] in the renal patient to assess suspected scar. Analysis was performed on the cMRF maps in the slice showing the greatest area of enhancement as defined on LGE. To determine if there are differences in tissue property values between scar and remote myocardium, ROIs were drawn on each of the maps in regions of normal appearing myocardium and areas of enhancement (defined by LGE where available or in regions of suspected scar defined by elevated *T*_1_ and *T*_1ρ_ values) and evaluated by an advanced imaging cardiologist. Voxel‐wise tissue property measurements were used to train several linear discriminant analysis (LDA) classifiers to assess whether these tissue property values could be used to differentiate between scar and remote myocardium. LDA models were trained using three sets of tissue properties: (1) *T*_1_ and *T*_2_ from conventional cMRF, (2) *T*_1f_ and *T*_2f_ from MT‐cMRF, and (3) BPF and *T*_2f_ from MT‐cMRF. Model performance was evaluated using a leave‐one‐out cross‐validation approach, where values from all but one patient were used to train the classifier and testing was conducted on the left‐out patient. This process was repeated for each patient resulting in five LDA classifiers for each set of tissue properties (15 in total). Classification accuracy was used as a metric to evaluate how different these tissue property values were between scar and normal tissue.

## 3 Results

### 3.1 Simulations

Measured *T*_1_/*T*_1f_, *T*_2_/*T*_2f_, and BPF and RMSE values for each simulated case are presented in Table 1 (see Figure S3 for maps). MT‐cMRF resulted in the lowest RMSE for both cases, whereas using a dictionary generated with conventional modeling substantially underestimated *T*_1_ values.

**TABLE 1:** Measured tissue property and RMSE values for simulations without (left) and with (right) MT modeling.

	Conventional cMRF		MT‐cMRF	
	Mean ± SD	RMSE	Mean ± SD	RMSE
Case 1: *T*_1_/*T*_1f_	827 ± 4 ms	17.3%	1001 ± 4 ms	0.3%
Case 1: *T*_2_/*T*_2f_	42.9 ± 0.1 ms	2.4%	44.0 ± 0.1 ms	0.2%
Case 1: BPF	—	—	8.0% ± 0.1%	1.0%
Case 2: *T*_1_/*T*_1f_ (H)	820 ± 3 ms	18.1%	994 ± 3 ms	0.6%
Case 2: *T*_2_/*T*_2f_ (H)	42.8 ± 0.1 ms	2.8%	43.9 ± 0.1 ms	0.3%
Case 2: BPF (H)	—	—	7.8% ± 0.1%	2.6%
Case 2: *T*_1_/*T*_1f_ (F)	895 ± 13 ms	18.7%	1064 ± 7 ms	3.3%
Case 2: *T*_2_/*T*_2f_ (F)	48.3 ± 1.9 ms	4.2%	49.5 ± 0.4 ms	0.9%
Case 2: BPF (F)	—	—	5.3% ± 0.2%	12.3%

### 3.2 Phantom Study

**FIGURE 2:**
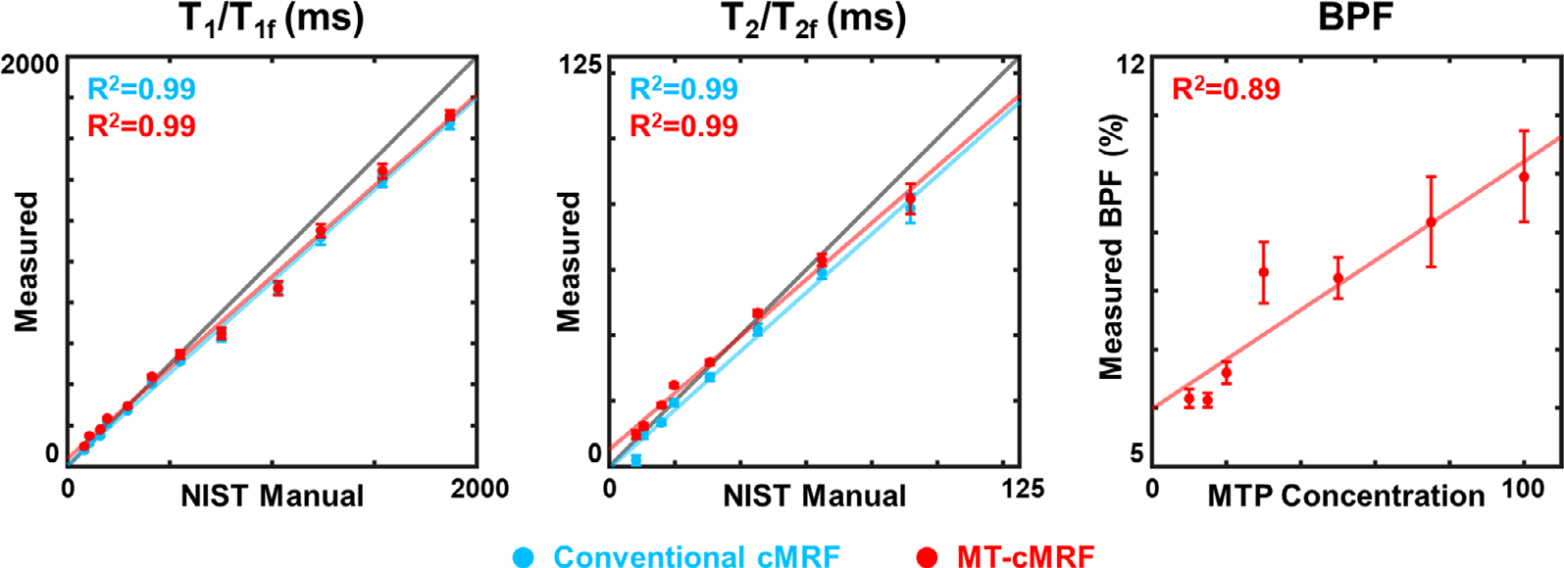
Correlation plots of the *T*_1_/*T*_1f_ (left), *T*_2_/*T*_2f_ (middle), and BPF (right) values measured using conventional cMRF (blue) and MT‐cMRF (red) compared to the NIST reference values for *T*_1_ and *T*_2_, and ground truth concentration of MTP for BPF. The gray line indicates unity and error bars show the SD over all pixels in each sphere.

Figure 2 shows *T*_1_/*T*_1f_ and *T*_2_/*T*_2f_ values measured in the ISMRM/NIST phantom and BPF in the MT phantom. Both conventional and MT‐cMRF show good agreement with reference values in the ISMRM/NIST phantom (*R* ^2^ = 0.99 for both). MT‐cMRF showed a linear relationship (*R* ^2^ = 0.89) between measured BPF and the concentration of MT material.

### 3.3 In Vivo Studies

#### 3.3.1 Healthy Subjects

**FIGURE 3:**
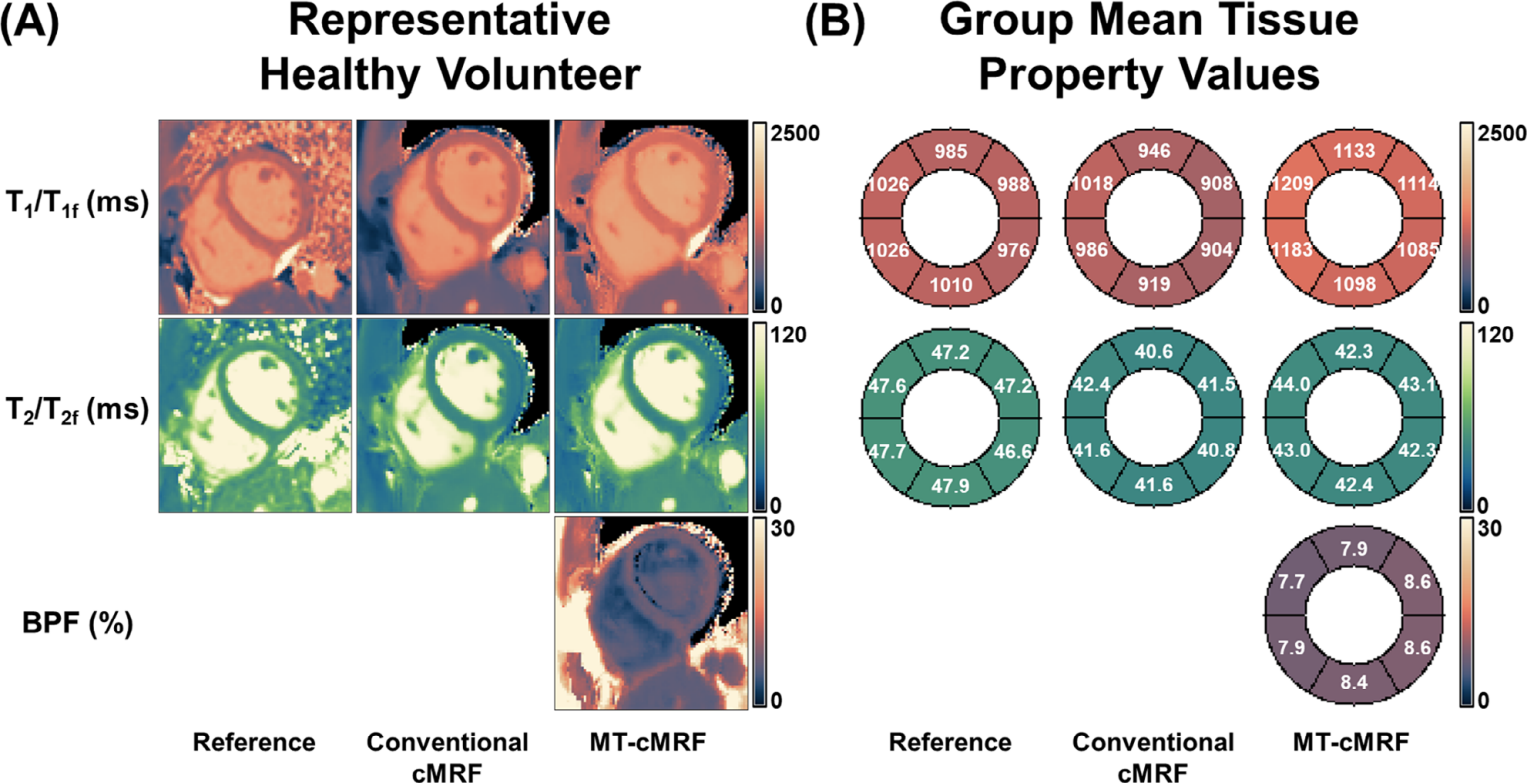
(A) *T*_1_/*T*_1f_ (top), *T*_2_/*T*_2f_ (middle), and BPF (bottom) maps from a single representative healthy subject measured using the reference sequence (left) and cMRF reconstructed with signal modeling that ignores (middle) and includes (right) MT effects. (B) Bullseye plots showing mean values measured in the six AHA segments across all 14 healthy volunteers. Note the higher measured values of *T*_1f_ and *T*_2f_ when MT is included in the model.

*T*_1_/*T*_1f_, *T*_2_/*T*_2f_, and BPF maps from reference sequences and cMRF reconstructed with dictionaries both ignoring and including MT processes from a representative subject are shown in Figure 3A. Corresponding mean *T*_1_/*T*_1f_, *T*_2_/*T*_2f_, and BPF values within the six AHA segments across all 14 volunteers are shown in Figure 3B. Group mean measurements for each method (reference/conventional cMRF/MT‐cMRF) were 1002 ± 44 ms/947 ± 75 ms/1137 ± 72 ms for *T*_1_/*T*_1f_, 47.4 ± 2.7 ms/41.5 ± 3.9 ms/42.8 ± 4.2 ms for *T*_2_/*T*_2f_, and 8.2% ± 1.2% for BPF. These values are consistent with previous studies (*T*_1_: [1030‐1250 ms], *T*_2_: [34‐55 ms], BPF: [3.1%–10.9%]) [15, 26, 27, 31]. A significant increase in *T*_1f_ and *T*_2f_ values was observed when including MT in the signal model compared to the *T*_1_ and *T*_2_ values measured without MT modeling (Myocardial Δ*T*_1_ = 190 ms, Δ*T*_2_ = 1.2 ms, *p* < 0.001 for both). Similar to prior cMRF findings [20], small differences with reference maps were observed for both conventional cMRF and MT‐cMRF, however, Bland–Altman analyses show agreement within 95% LoA (see SI: *Healthy Subject Analyses* for comparison).

#### 3.3.2 Patients

**FIGURE 4:**
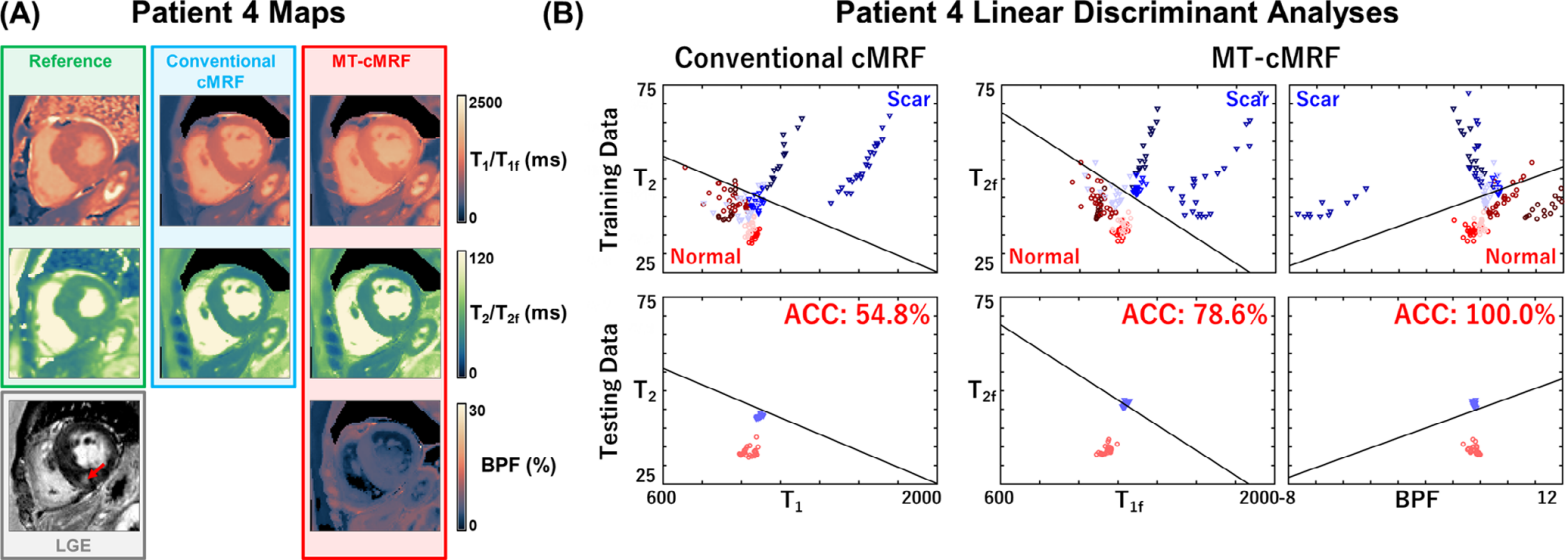
(A) *T*_1_/*T*_1f_ (top), *T*_2_/*T*_2f_ (middle), and BPF (bottom‐right) maps for a single patient derived from the cMRF data reconstructed using a signal model that ignores (middle) and includes MT effects (right). The corresponding reference maps (left) and LGE image (bottom‐left). Red arrow indicates regions of scar. (B) Linear discriminant analysis (LDA) between voxels in regions of scar (blue) and normal appearing myocardium (red) using conventional modeling (i.e., ignoring MT effects) *T*_1_ and *T*_2_ (left), MT modeling using *T*_1f_ and *T*_2f_ (middle), and *T*_2f_ and BPF (right). Accuracy for testing data prediction is reported in each plot. The different shadings of each color indicate values from different patients.

Figure 4A shows *T*_1_/*T*_1f_, *T*_2_/*T*_2f_, and BPF maps reconstructed using cMRF dictionaries both ignoring and including MT processes along with the corresponding reference maps and LGE for a representative patient. Figure 4B shows the corresponding LDA decision boundaries determined by training on the other patients and testing on this patient for each of the three tissue property sets: (1) *T*_1_ and *T*_2_ from conventional cMRF, (2) *T*_1f_ and *T*_2f_ from MT‐cMRF, and (3) BPF and *T*_2f_ from MT‐cMRF. (See SI: *Patient Analyses* for remaining patient maps and LDA plots) Across all models, conventional cMRF yielded an average out‐of‐sample predictive accuracy of 61.8%, whereas MT‐cMRF accuracy was 82.4% when using *T*_1f_/*T*_2f_ as the tissue properties to distinguish between scar and normal appearing, myocardium, and 86.1% when using *T*_2f_/BPF.

## 4 Discussion

This study demonstrates that (1) significantly different values of *T*_1_ and *T*_2_ are measured with cMRF when ignoring and including the effects of MT in the signal model, and (2) when including MT effects in the signal model, tissue properties measured using MRF provide improved differentiation between regions of apparent scar and healthy myocardium. The MT‐cMRF approach was first evaluated in simulation and phantom studies to assess the accuracy of measurements compared to conventional modeling. This study showed that MT‐cMRF yields more accurate *T*_1(f)_ and *T*_2(f)_ measurements than when using conventional signal modeling in simulation, and includes the added capability of mapping the BPF by simply including MT effects in the signal model. MT‐cMRF was then deployed in healthy subjects to assess whether the inclusion of MT effects alters the resulting measurements of *T*_1_ and *T*_2_. A significant increase in *T*_1f_ and *T*_2f_ values was observed when modeling MT in comparison to cMRF modeling which ignores these effects in healthy subjects. Finally, tissue property maps were generated using both cMRF and MT‐cMRF in patients with LGE (or elevated *T*_1ρ_) to assess differences in myocardial values between remote and scarred myocardium. The inclusion of MT effects in the signal model improved the ability of an LDA classifier to distinguish between healthy and scarred tissue, indicating differences in native tissue property values between these regions.

MT modeling was first introduced into the MRF framework by Hilbert et al. [8], who found that ignoring MT effects led to decreases in *T*_1_ and *T*_2_ measurements made with the conventional MRF acquisition in the brain relative to those which included MT effects using a two‐pool modeling scheme. This study also showed for the first time that the BPF could be mapped using MRF by moving from a single pool model to a two‐pool model which includes the effects of MT. West et al. [32] used MRF to investigate the effects of inhomogeneous MT and demonstrated that MRF acquisition parameters and modeling can be tailored to specifically highlight different macromolecular‐based properties, ultimately enabling more detailed and complete tissue characterization. These initial findings highlight the potential for advanced MRF methods to be tailored for nuanced evaluation of macromolecular content and exchange in different tissues. This study builds on and extends these publications by investigating the use of MT modeling in cardiac tissue characterization with cMRF. The findings presented here closely align with these initial observations in the brain (i.e., that ignoring MT effects results in lower *T*_1_ and *T*_2_ estimates and including MT modeling enables quantification of the BPF), suggesting that this approach is equally applicable to cardiac tissue.

Few studies have explored the use of MT for the evaluation of cardiac tissue, and the properties measured among those that have are highly variable. López et al. [15] measured the pool size ratio (PSR) in the myocardium by matching a series of MT‐weighted images to a signal model dictionary and found decreased PSR values in regions of LGE enhancement. Similarly, López et al. [14] measured the MTR using two image acquisitions, with and without off‐resonance MT preparations prior to imaging, and showed that the MTR was lower in regions of scar compared to remote myocardium. Duan et al. [11] introduced an MT‐corrected *T*_1_ mapping technique which resulted in elevated *T*_1_ values compared to non‐corrected measurements, and demonstrated improved myocardium‐to‐scar contrast‐to‐noise ratio compared to conventional MOLLI mapping. The findings presented here are consistent with these works showing decreased BPF in regions of enhancement, as well as increased *T*_1_ values when modeling MT effects. Additionally, elevated *T*_2_ was observed in regions of scar, highlighting the potential value of multiparametric evaluation for distinguishing between normal and scarred tissue.

To the best of our knowledge, this is the first study to apply MRF with explicit modeling of MT in the heart, providing new insights into the utility of cMRF for cardiac tissue characterization. It is well established that cMRF, much like other conventional quantification techniques such as MOLLI, tends to underestimate *T*_1_ and *T*_2_ values [7, 9]. The results presented here demonstrate that integrating MT modeling into the cMRF framework reduces this underestimation. In particular, the *T*_1f_ values measured using MT‐cMRF are in the range of those obtained with SASHA (1144 ± 45 ms) [31], which is known to be less susceptible to MT effects and therefore thought to be more accurate. Notably, this study also shows that including MT in the cMRF signal model improves the utility of native tissue properties to discriminate between scarred and remote myocardium, suggesting that MT‐cMRF may be useful for contrast‐free assessment of cardiac disease.

There are several limitations in this study. First, the MT modeling approach relies on several commonly‐used assumptions, including fixing the free and bound pool *T*_1_ values to be equal (*T*_1f_ = *T*_1b_) and using constant values for the exchange rate (*k*_f_) and bound pool *T*_2_ (*T*_2b_), rather than utilizing a fully quantitative MT model. Note that these assumptions have been made by multiple groups studying MT quantification [8, 11, 15, 24, 33, 34]. While these assumptions improved computational efficiency by reducing the number of parameters to be modeled in the dictionary and enabled practical implementation in this initial study, recent studies [12, 13] have shown that such constraints may not be valid and utilizing a fully quantitative model may provide additional insight into tissue health. However, implementing a fully quantitative model would substantially increase computational demands, not only because of the greater model complexity but also due to the memory required to account for variability in up to six parameters. Moreover, the simplified three‐parameter model produced tissue property values that are consistent with prior work, and future work will investigate the utility of a fully quantitative approach. Additionally, this study was conducted in a small cohort of patients where the final diagnosis was unknown. As a result, it is not possible to draw conclusions regarding the overall clinical utility of MT‐cMRF for scar detection or disease discrimination in larger and more diverse populations. Future studies will include a larger, well‐characterized patient cohort.

## 5 Conclusion

This study demonstrates that *T*_1_ and *T*_2_ measurements made with cMRF are influenced by MT and that explicitly modeling MT effects elevated measurements made in simulation and in vivo. Additionally, MT‐cMRF enabled direct mapping of the BPF through straightforward inclusion of MT in the signal model, adding potentially valuable macromolecular information without lengthening the scan. In patients, the tissue property values measured with MT‐cMRF were better able to differentiate between normal‐appearing myocardium and scarred regions compared to measurements made using conventional models.

## Funding

This work was supported by the National Institute on Aging (R01AG059729), Siemens Healthineers, National Institute of Diabetes and Digestive and Kidney Diseases (U01DK123013), Division of Cancer Prevention, National Cancer Institute (R01 CA 263583), American Heart Association (20SFRN35120123), and National Heart, Lung, and Blood Institute (R01 HL153034, R01 HL163030, R01 HL163991, R01HL136685).

## Conflicts of Interest

Venkatesh Murthy owns stock in General Electric, Cardinal Health, Viatris, Pfizer, Amgen, Merck and Johnson & Johnson and stock options in Ionetix. He is a paid consultant for INVIA Medical Imaging Solutions & Siemens Healthineers. Venkatesh Murthy has received research support through his institution from Siemens Healthineers. Venkatesh Murthy is supported by the Melvyn Rubenfire Professorship in Preventive Cardiology. Venkatesh Murthy is also supported by R01AG059729, R01HL136685, U01DK123013 from the National Institutes of Health and AHA Strategically Focused Research Network 20SFRN35120123. Nicole Seiberlich has received royalties from Siemens Healthineers for MRF. Nicole Seiberlich and Jesse Hamilton receive research support from Siemens Healthineers.

## Supporting information


**Text S1:** Reconstruction Comparison.
**Figure S1:** (A) *T*_1f_, *T*_2f_, and BPF maps from a single representative healthy subject measured using MT‐cMRF reconstructed with an SVD + direct matching (left), low‐rank (middle), and DIP (right) approach. (B) Bullseye plots showing the mean and (C) COV for the *T*_1f_, *T*_2f_, and BPF values measured in the six AHA segments across all 14 healthy volunteers.
**Text S2:** MT Encoding in the cMRF Fingerprints.
**Figure S2:** (A) Simulated signal evolutions that ignore (blue) and include (red) MT in the signal model. The absolute difference between the signals is plotted in black. (B) Simulated signal evolutions for various BPF values. All signal evolutions use a typical myocardial voxel (*T*_1_/*T*_1f_ = 1000 ms, *T*_2_/*T*_2f_ = 44 ms).
**Text S3:** XCAT simulations.
**Figure S3:** XCAT phantom experiments simulating (A) a healthy heart and (B) a heart with a scar (indicated by the red arrow). Ground truth (left) tissue property maps are compared to those generated cMRF reconstructed with conventional (middle) and MT modeling (right). RMSE values for healthy (H) and fibrotic (F) myocardium are reported.
**Table S1:** Measured tissue property and RMSE values for simulations with various heart rates.
**Text S4:** MT Phantom Preparation.
**Text S5:** Healthy Subject Analyses.
**Figure S4:** Bland–Altman plots comparing conventional cMRF (left)and MT‐cMRF (right) with reference values for *T*_1_ (top) and *T*_2_ (bottom) for all healthy volunteers. On each plot, bias is indicated by the solid line, and dashed lines indicate the 95% limits of agreement.
**Text S4:** Patient Analyses.
**Figure S5:** *T*_1_/*T*_1f_ (top), *T*_2_/*T*_2f_ (middle), and BPF (bottom‐right) maps for each patient derived from the cMRF data reconstructed using a signal model that ignores (center) and includes MT effects (right) along with the corresponding reference maps and LGE image or *T*_1ρ_ map (left). Patient scans referred for cardiomyopathy (purple), renal impairment (yellow), and HCM (pink) are indicated by outline color. Red arrows indicate regions of scar.
**Figure S6:** Leave‐one‐out linear discriminant analysis (LDA) between voxels in regions of scar (blue) and normal appearing myocardium (red) using (A) conventional modeling (i.e., ignoring MT effects) *T*_1_ and *T*_2_, (B) MT modeling *T*_1f_ and *T*_2f_, and (C) MT modeling *T*_2f_ and BPF. Accuracy for testing data prediction is reported in each plot. The different shadings of each color indicate values from different patients.
**Figure S7:** *T*_1_/*T*_1f_ (top), *T*_2_/*T*_2f_ (middle), and BPF (bottom) values measured using conventional cMRF (left) and MT‐cMRF (right) in both the healthy‐appearing myocardium and in areas of scar in the patients. Significant differences are indicated as ** (*p* < 0.01).

## Data Availability

The data that support the findings of this study are available on request from the corresponding author. The data are not publicly available due to privacy or ethical restrictions. EPG‐X modeling code is available at http://www.github.com/mriphysics/EPG‐X.
