# Supplementary material for: A Comparison of Tissue Property Values Estimated Using Conventional Cardiac MRF and MT‐Cardiac MRF
Source: Magn Reson Med. 2026 Jul 1;96(4):1806–13. doi: 10.1002/mrm.70470 (PMC13419325; doi:10.1002/mrm.70470)
Supplement: Supplementary file 1 — Text S1: Reconstruction Comparison. Figure S1: (A) T 1f, T 2f, and BPF maps from a single representative healthy subject measured using MT‐cMRF reconstructed with an SVD + direct matching (left), low‐rank (middle), and DIP (right) approach. (B) Bullseye plots showing the mean and (C) COV for the T 1f, T 2f, and BPF values measured in the six AHA segments across all 14 healthy volunteers. Text S2: MT Encoding in the cMRF Fingerprints. Figure S2: (A) Simulated signal evolutions that ignore (blue) and include (red) MT in the signal model. The absolute difference between the signals is plotted in black. (B) Simulated signal evolutions for various BPF values. All signal evolutions use a typical myocardial voxel (T 1/T 1f = 1000 ms, T 2/T 2f = 44 ms). Text S3: XCAT simulations. Figure S3: XCAT phantom experiments simulating (A) a healthy heart and (B) a heart with a scar (indicated by the red arrow). Ground truth (left) tissue property maps are compared to those generated cMRF reconstructed with conventional (middle) and MT modeling (right). RMSE values for healthy (H) and fibrotic (F) myocardium are reported. Table S1: Measured tissue property and RMSE values for simulations with various heart rates. Text S4: MT Phantom Preparation. Text S5: Healthy Subject Analyses. Figure S4: Bland–Altman plots comparing conventional cMRF (left)and MT‐cMRF (right) with reference values for T 1 (top) and T 2 (bottom) for all healthy volunteers. On each plot, bias is indicated by the solid line, and dashed lines indicate the 95% limits of agreement. Text S4: Patient Analyses. Figure S5: T 1/T 1f (top), T 2/T 2f (middle), and BPF (bottom‐right) maps for each patient derived from the cMRF data reconstructed using a signal model that ignores (center) and includes MT effects (right) along with the corresponding reference maps and LGE image or T 1ρ map (left). Patient scans referred for cardiomyopathy (purple), renal impairment (yellow), and HCM (pink) are indicated by outline color. Red arro [file MRM-96-1806-s001.docx]

**Supplemental Material**

**Reconstruction Comparison**

T_1f_, T_2f_, and BPF maps reconstructed using SVD+direct matching, low-rank, and DIP methods are shown in SI Fig. 1A for a representative healthy subject. Myocardial T_1f_, T_2f_, and BPF values were obtained by computing the mean and SD within manually drawn ROIs using the six mid-ring American Heart Association (AHA) segments^30^ and the coefficient of variation (COV) was computed for each ROI and compared between reconstruction methods. Mean T_1f_, T_2f_, and BPF values and their corresponding COV from ROIs drawn in the six AHA segments for each healthy volunteer are shown in the bullseye plots in SI Fig. 1B,C, respectively. Similar to prior studies^20 – 23^, DIP reduced the COV across all segments compared to the other two reconstruction methods.


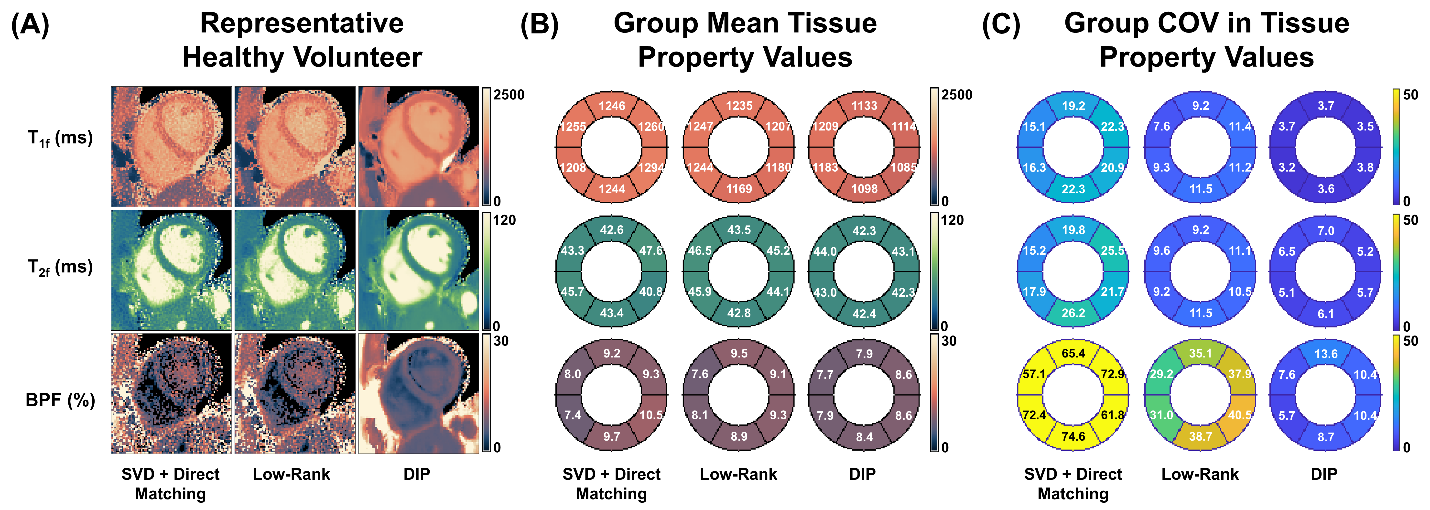


**Supplementary Figure 1.** (A) T_1f_, T_2f_, and BPF maps from a single representative healthy subject measured using MT-cMRF reconstructed with an SVD+direct matching (left), low-rank (middle), and DIP (right) approach. (B) Bullseye plots showing the mean and (C) COV for the T_1f_, T_2f_, and BPF values measured in the six AHA segments across all 14 healthy volunteers.

**MT Encoding in the cMRF Fingerprints**

Signal evolutions for the same relaxation parameters (T_1_/T_1f_=1000ms, T_2_/T_2f_=44ms) using modeling that both ignores and includes MT effects are shown in SI Fig. 2A. The largest differences were observed after the heartbeats that contained preparation pulses, where inversions resulted in larger differences than T_2_-preps. Signal evolutions for the same relaxation parameters but with different BPF sizes are shown in SI Fig. 2B to demonstrate separability between MT parameters using the described cMRF sequence.


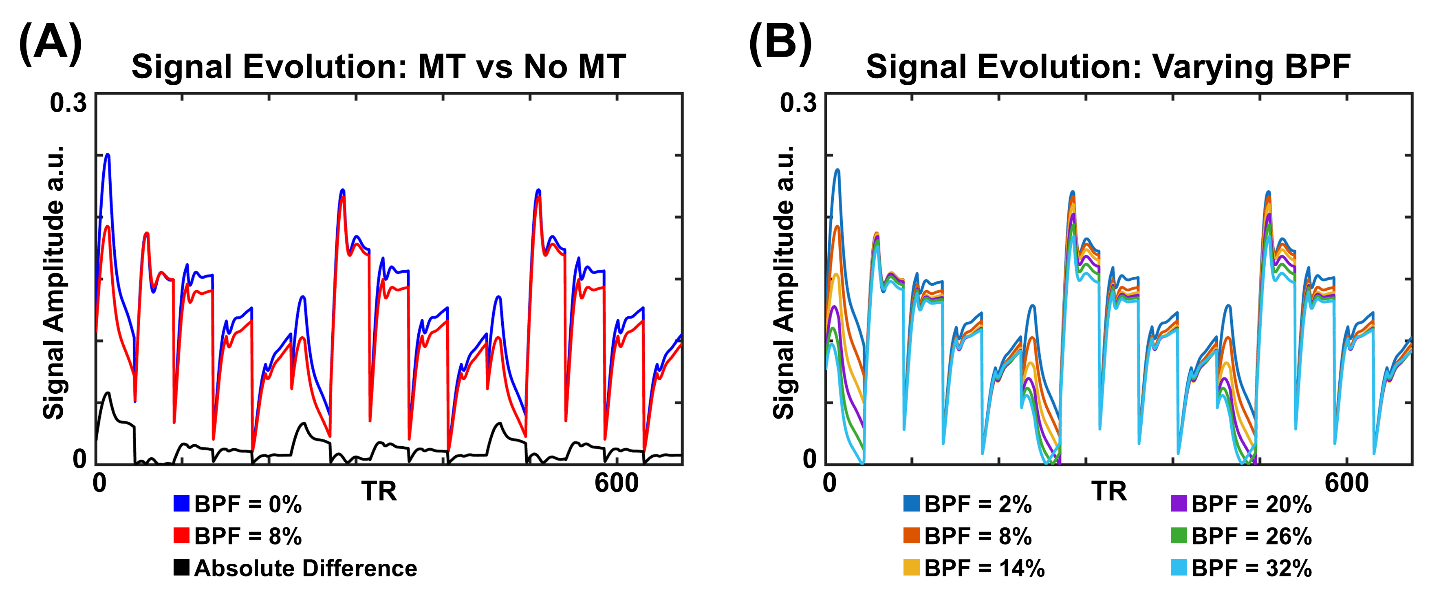


**Supplementary Figure 2.** (A) Simulated signal evolutions that ignore (blue) and include (red) MT in the signal model. The absolute difference between the signals is plotted in black. (B) Simulated signal evolutions for various BPF values. All signal evolutions use a typical myocardial voxel (T_1_/T_1f_=1000ms, T_2_/T_2f_=44ms).

**XCAT Simulations**

SI Fig. 3 shows the ground truth T_1_/T_1f_, T_2_/T_2f_, and BPF maps as well as the cMRF maps generated using both conventional and MT modeling for the simulated cases of healthy myocardium and myocardium with a small lesion.


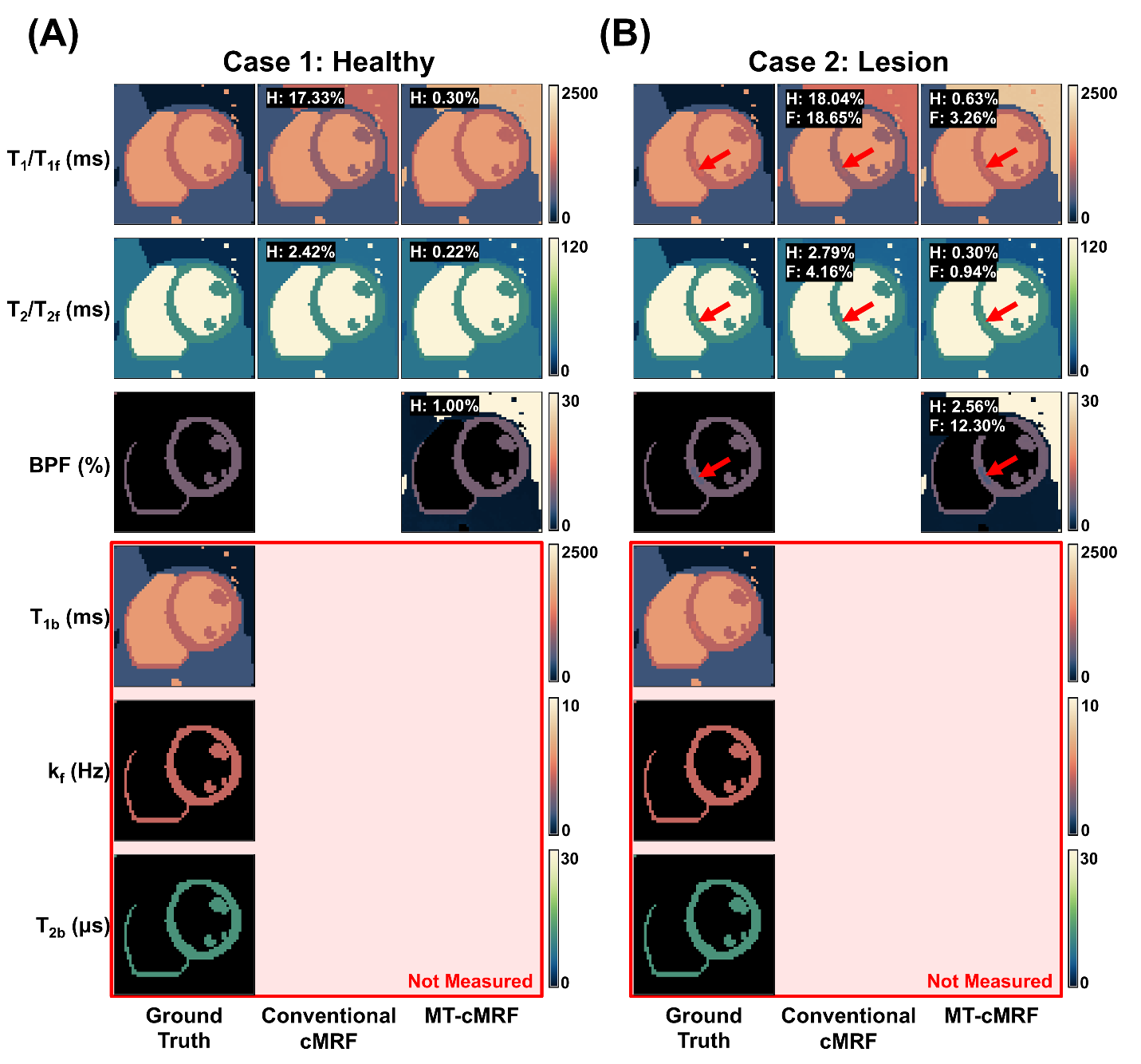


**Supplementary Figure 3.** XCAT phantom experiments simulating (A) a healthy heart and (B) a heart with a scar (indicated by the red arrow). Ground truth (left) tissue property maps are compared to those generated cMRF reconstructed with conventional (middle) and MT modeling (right). RMSE values for healthy (H) and fibrotic (F) myocardium are reported.

Simulations using the XCAT phantom^25^ with a simulated heart rate from 40-100bpm were conducted for the same relaxation parameters (T_1f_=1000ms, T_2f_=44ms, and BPF=8%) in the healthy myocardium case to assess the effect of heart rate on MT encoding. We found similar performance in tissue property values obtained across each HR defined by the RMSE. Mean tissue property values and RMSE for each heart rate are shown in SI Table 1.

|  | **40 bpm** | **60 bpm** | **80 bpm** | **100 bpm** |
| --- | --- | --- | --- | --- |
| **Case 1: T_1f_** | | | | |
| **Mean±SD** | 989±7ms | 1001±4ms | 988±3ms | 998±2ms |
| **RMSE** | 1.1% | 0.3% | 1.2% | 0.2% |
| **Case 1: T_2f_** | | | | |
| **Mean±SD** | 44.1±0.1ms | 44.0±0.1ms | 44.0±0.2ms | 44.0±0.2ms |
| **RMSE** | 0.3% | 0.2% | 0.2% | 0.3% |
| **Case 1: BPF** | | | | |
| **Mean±SD** | 7.8±0.2% | 8.0±0.1% | 7.9±0.1% | 8.0±0.1% |
| **RMSE** | 2.2% | 1.0% | 1.3% | 0.7% |

**Supplementary Table 1.** Measured tissue property and RMSE values for simulations with various heart rates.

**MT Phantom Preparation**

A Cardiac MT phantom (MTP) was made with a 200 uM Mn solution, cetearyl alcohol (CA), and behentrimonium chloride (BTAC). 20g CA and 5 g BTAC were heated together in a microwave until melted. 200ml of 200uM Mn in deionized water was heated in a microwave to ~85°C. The hot Mn solution was placed on a hot plate/stirrer and set to 90°C with mild stirring. The molten CA/BTAC was slowly poured into the hot water. The solution was covered and temperature reduced to 60°C. The mixture held at 60°C for 30 minutes, allowing formation of a lamellar gel network (LGN) from water/CA/BTAC. This sample (called 100% MTP) has a proton mole fraction of from water of approximately 86% and from the semisolids of about 14%.

To make MTP materials of different concentrations, appropriate amounts of 100% MTP were diluted with 200 uM Mn, heated to ~85°C, cooled to 60°C as above to form LGN, transferred into 25ml scintillation vials, and incorporated into the MT phantom array.

**Healthy Subject Analyses**

SI Fig. 4 shows the Bland-Altman analysis between reference maps and cMRF measurements. For T_1_, conventional cMRF had a bias of −54 with LoA (−117, 8) ms and MT-cMRF had a bias of 136 with LoA (74, 199) ms. For T_2_, conventional cMRF had a bias of -5.9 with LoA (−11.9, 0.0) ms and MT-cMRF had a bias of −4.6 with LoA (−11.0, 1.8) ms.


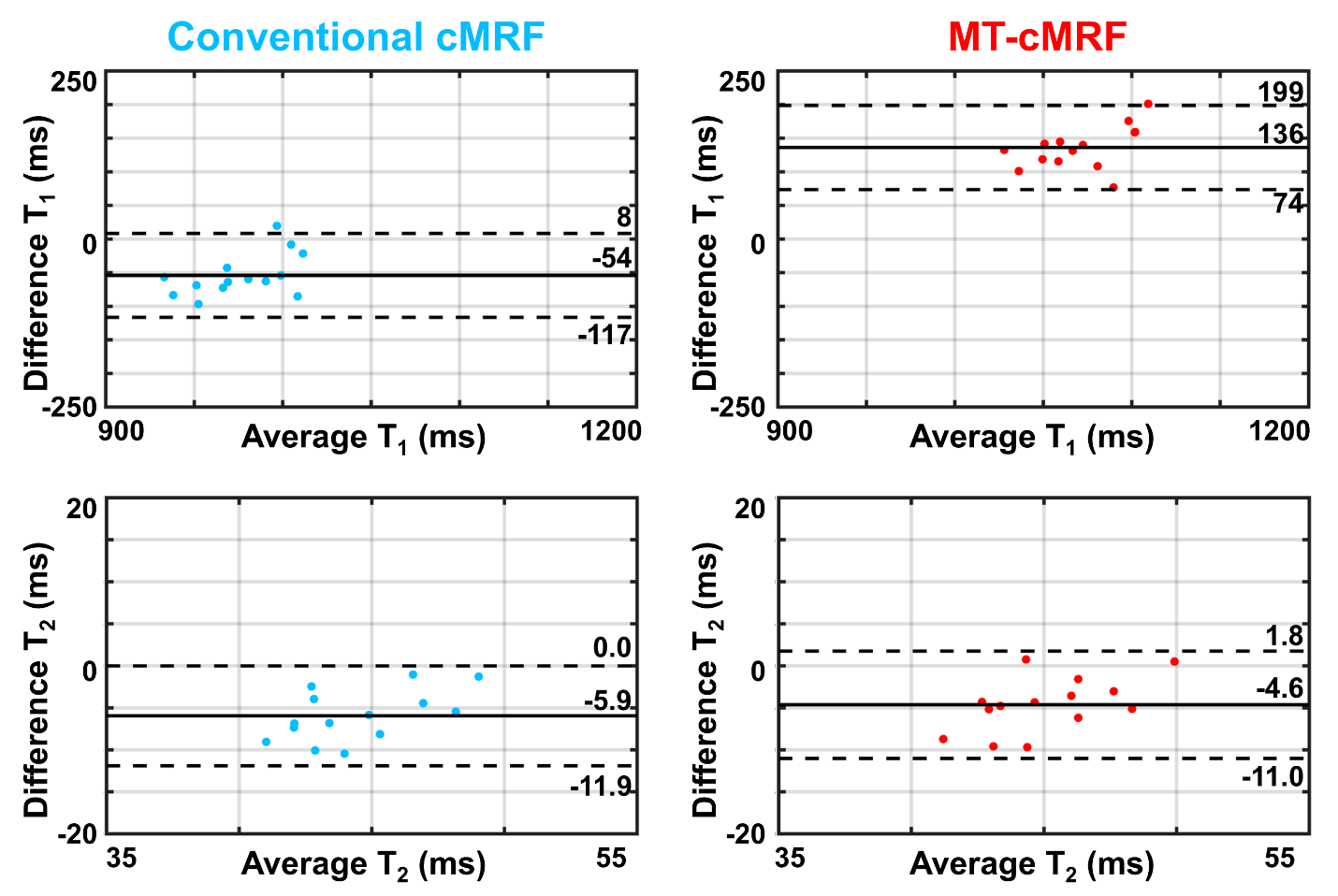


**Supplementary Figure 4.** Bland–Altman plots comparing conventional cMRF (left)and MT-cMRF (right) with reference values for T_1_ (top) and T_2_ (bottom) for all healthy volunteers. On each plot, bias is indicated by the solid line, and dashed lines indicate the 95% limits of agreement.

**Patient Analyses**

T_1_/T_1f_, T_2_/T_2f_, and BPF maps reconstructed using cMRF dictionaries both ignoring and including MT processes along with the corresponding reference maps and LGE slice are shown for all patients in SI Fig. 5.


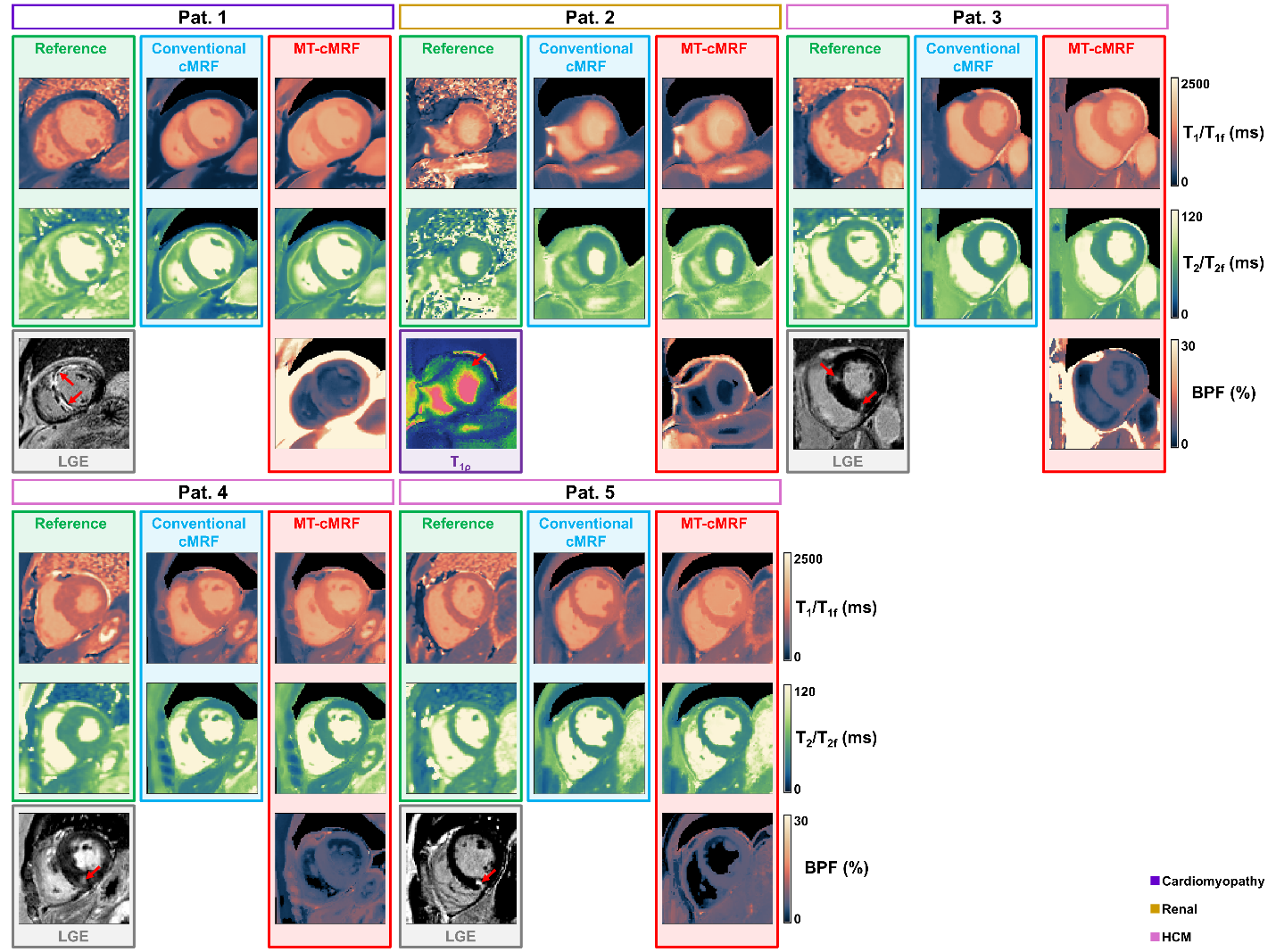


**Supplementary Figure 5.** T_1_/T_1f_ (top), T_2_/T_2f_ (middle), and BPF (bottom-right) maps for each patient derived from the cMRF data reconstructed using a signal model that ignores (center) and includes MT effects (right) along with the corresponding reference maps and LGE image or T_1ρ_ map (left). Patient scans referred for cardiomyopathy (purple), renal impairment (yellow), and HCM (pink) are indicated by outline color. Red arrows indicate regions of scar.

SI Fig. 6 shows the decision boundary plots from each LDA classification model for both the training and testing datasets.


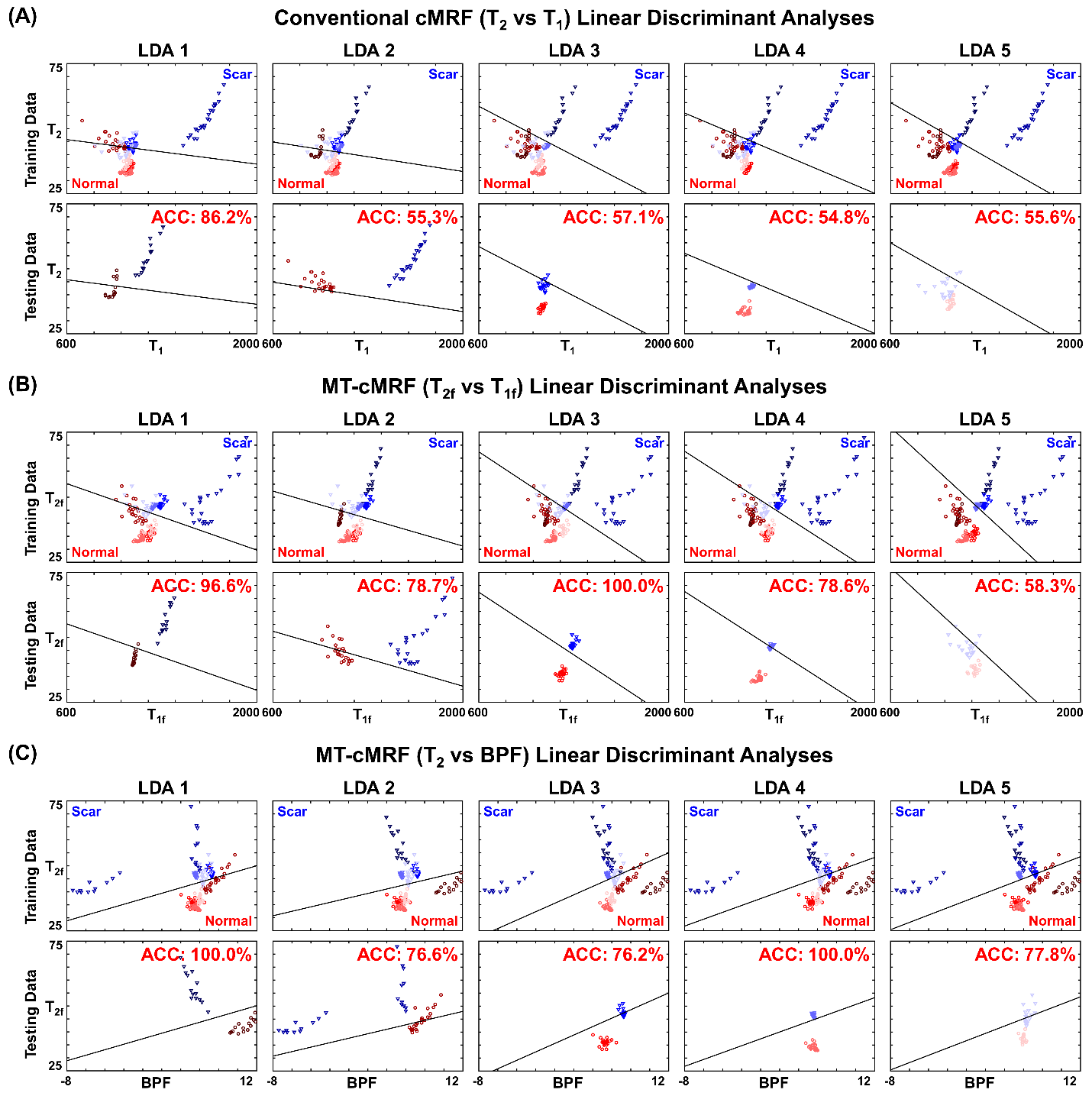


**Supplementary Figure 6.** Leave-one-out linear discriminant analysis (LDA) between voxels in regions of scar (blue) and normal appearing myocardium (red) using (A) conventional modeling (i.e. ignoring MT effects) T_1_ and T_2_, (B) MT modeling T_1f_ and T_2f_, and (C) MT modeling T_2f_ and BPF. Accuracy for testing data prediction is reported in each plot. The different shadings of each color indicate values from different patients.

SI Fig 7 shows mean T_1_/T_1f_, T_2_/T_2f_, and BPF values from ROIs drawn within regions of normal-appearing myocardium (T_1_=1008±59ms, T_2_=38.4±4.7ms for conventional modeling; T_1f_=1154±59ms, T_2f_=38.9±4.3ms, BPF=7.1±2.5% for MT modeling) and scar (T_1_=1198±245ms, T_2_=47.5±6.0ms for conventional modeling; T_1f_=1338±191ms, T_2f_=49.4±4.6ms, BPF=5.2±2.8% for MT modeling) for each patient. Paired t-test analysis showed no difference in T_1_ (p=0.22), T_1f_ (p=0.15), and BPF (p=0.33) in regions of scar relative to normal-appearing myocardium and a significant increase in T_2_ and T_2f_ values (p=0.001 and 0.004 respectively).


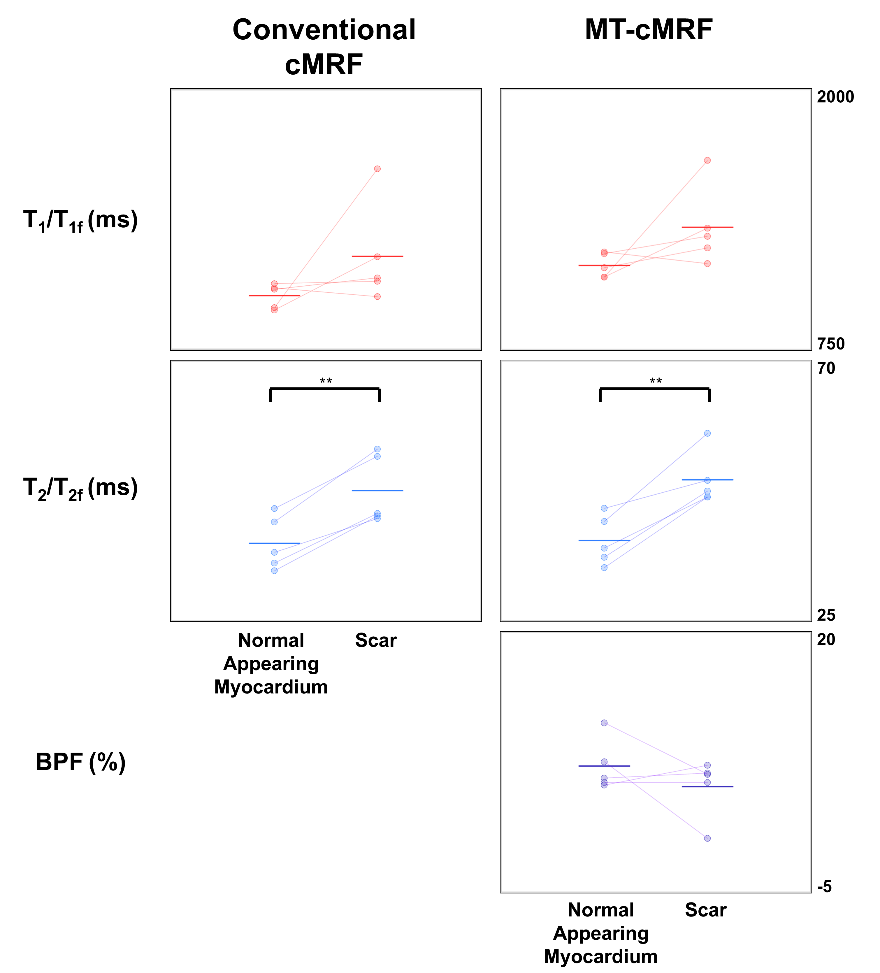


**Supplementary Figure 7.** T_1_/T_1f_ (top), T_2_/T_2f_ (middle), and BPF (bottom) values measured using conventional cMRF (left) and MT-cMRF (right) in both the healthy-appearing myocardium and in areas of scar in the patients. Significant differences are indicated as ** (p < 0.01).
